# Performance analysis: Securing SIP on multi-threaded/multi-core proxy server using public keys on Diffie–Hellman (DH) in single and multi-server queuing scenarios

**DOI:** 10.1371/journal.pone.0293626

**Published:** 2024-01-25

**Authors:** David Samuel Bhatti, Salbia Sidrat, Shahzad Saleem, Annas Wasim Malik, BeomKyu Suh, Ki-Il Kim, Kyu-Chul Lee

**Affiliations:** 1 Department of Computer Science, University of Central Punjab, Lahore, Pakistan; 2 Department of Computer Science, The Institute of Management Sciences, Lahore, Pakistan; 3 Department of Cybersecurity, College of Computer Science and Engineering, Jeddah University, Jeddah, KSA; 4 School of Electrical Engineering and Computer Science, National University of Sciences and Technology, Islamabad, Pakistan; 5 Department of Computer Science and Engineering, Chungnam National University, Daejeon, Republic of Korea; Jaramogi Oginga Odinga University of Science and Technology, KENYA

## Abstract

The rapid replacement of PSTN with VOIP networks indicates the definitive phase-out of the PBX/PABX with smartphone-based VOIP technology that uses WLAN connectivity for local communication; however, security remains a key issue, regardless of the communication coverage area. Session initiation protocol (SIP) is one of the most widely adopted VOIP connection establishment protocols but requires added security. On the Internet, different security protocols, such as HTTPS (SSL/TLS), IPSec, and S/MIME, are used to protect SIP communication. These protocols require sophisticated infrastructure and some pose a significant overhead that may deteriorate SIP performance. In this article, we propose the following: i) avoid using Internet bandwidth and complex Internet protocols for local communication within an organization, but harness WLAN connectivity, ii) use multi-threaded or multicore computer systems to handle concurrent calls instead of installing hardware-based SIP servers, and iii) run each thread in a separate core. Cryptography is a key tool for securely transmitting confidential data for long- and short-range communication, and the Diffie-Hellman (DH) protocol has consistently been a popular choice for secret key exchanges. Primarily, used for symmetric key sharing, it has been proven effective in generating public/private key pairs, sharing public keys securely over public channels, and subsequently deriving shared secret keys from private/public keys. This key exchange scheme was proposed to safeguard VOIP communication within WLANs, which rely on the SIP for messaging and multimedia communication. For ensuring an efficient implementation of SIP, the system was rigorously analyzed using the M/M/1 and M/M/c queuing models. We analyze the behavior of SIP servers with queuing models with and without end-to-end security and increase users’ trust in SIP security by providing a transparent sense of end-to-end security as they create and manage their private and public keys instead of relying on the underlying SIP technology. This research implements instant messaging, voice conversation, and secret key generation over DH while implementing and observing the role of multi-threading in multiqueue systems that serve incoming calls. By increasing the number of threads from one to two, the SIP response time improved from 20.23809 to 0.08070 min at an arrival rate of 4250 calls/day and a service rate of three calls/min. Similarly, by adding one to seven threads, the queue length was reduced by four calls/min. Implementing secure media streaming and reliable AES-based signaling for session confidentiality and integrity introduces a minor 8-ms tradeoff in SIP service performance. However, the advantages of implementing added security outweigh this limitation.

## 1 Introduction

Applications such as WhatsApp, Facebook, and VChat offer secure communication but depend entirely on Internet connectivity through cellular or satellite links. An alternative to PBX/PABX is VOIP, which uses internet protocol (IP) networks for text, image, and audio/video transmission. The purpose of this introspection is to explore the feasibility of implementing session initiation protocol (SIP) on a local network (WiFi) as opposed to relying on internet connectivity. We aim to provide a secure communication solution that operates independently of the Internet connectivity, reducing the dependence on bandwidth and enabling efficient communication within the local network environment. VOIP requires a signaling protocol to establish a connection between networks/devices, transmit data, and terminate the connection. Most VOIP applications support signaling protocols, such as session initiation protocol (SIP) and H.323 [1]. H.323 was developed by the ITU as a standard for routing between circuit-switched and packet-switched networks to tear down IP-originating calls to the PSTN. However, IP-based conversation has been rapidly gaining popularity [2]. SIP is a VOIP session creation, maintenance, and termination protocol that operates on the application layer of TCP/IP.

SIP is used as a signaling standard to initiate, terminate, and monitor interactive media sessions and is required for most VoIP communication [2, 3]. It has recently emerged as the dominant signaling protocol for Internet applications to implement various features, such as video conferencing, peer-to-peer communication, instant messaging, and voicemail. Mobile applications, which are more customizable than traditional applications, are also available in SIP. When SIP is utilized in a wide range of wired and wireless networks, security problems arise, usually determined by confidentiality, integrity, and availability (CIA) [4].

The massive rise of VoIP significantly impacts IP-based corporate applications that provide dozens of new real-time media services. Because of the reduced cost and higher quality, organizations are adopting various VoIP services, including voice conversations, text messages, and video conferencing, to increase their versatility.

This study aims to develop a cost-effective wireless local area network (WLAN)-based solution for secure VOIP communication using SIP as the signaling protocol. The focus is on implementing symmetric-key cryptography to ensure security without wired connections. An essential goal is to gain a comprehensive understanding of the SIP framework’s performance under various conditions, particularly with or without application-layer security, because SIP lacks inherent security when not using HTTPS (TLS/SSL) [5]. Hence, without these secure protocols, SIP communications can be vulnerable to security threats. HTTPS provides encryption and authentication, ensuring secure data transmission between clients and servers. Implementing HTTPS alongside SIP can enhance the overall security of multimedia communication and protect against potential security risks [6, 7]. Therefore, this study examined the performance and optimization of SIP using M/M/1 and M/M/c queuing models, commonly employed in telephony [8–11]. The M/M/c model involves a dedicated SIP proxy server with multiple threads that handle incoming calls from SIP endpoints, offering cost-effective multi-threading solutions. In addition, a dedicated intermediary server was employed to reduce delays between the SIP servers. This study analyzed critical performance parameters such as call wait time, queue length (number of calls in the wait state), and probability of server availability under different scenarios. The results demonstrate that SIP can be effectively applied using a single-server queuing system; however, for higher call rates (above 2500 calls/day), it is preferable to employ a multi-server queuing system. The M/M/c model with security protocols is recommended for call rates exceeding 2500 calls/day, whereas the M/M/1 model is suitable for other cases at a service rate of 3. In the worst-case scenario, the call rate should not exceed 4250 calls/day for M/M/1.

In this study, SIP is deployed using WiFi, which employs IP to establish communication between two local area devices; if provided with an Internet connection, it can connect two long-distance devices [12, 13]. Billions of people connect to different WiFi (IEEE 802.11) sites to connect to their gadgets, such as smart TVs, phones, and other Internet of Things devices [14, 15]. WiFi ensures distinct advantages such as mobility, reduced cost, lower budget, high scalability, simple infrastructure, easy installation, and the ability to expand the range of networks in an organization. The most prominent concern in WiFi 802.11 networks when SIP is applied is security, which was addressed using public-key cryptography in this study [16, 17].

### 1.1 Contributions

The salient contributions of this research are as follows:

1. The current research highlights a vulnerability in SIP, where it becomes ineffective when deployed in local and global environments without HTTPS (TLS/SSL). To address this issue, we propose enhancing security by implementing end-to-end encryption through symmetric key cryptography in WLAN environments.
2. It analyses the performance of SIP when an additional protection layer is provided as end-to-end encryption in two different queuing systems, namely M/M/1 and M/M/c. Implementing customized end-to-end security has a negligible impact on SIP performance. Furthermore, using AES for secure media streaming and signaling introduces only a minor tradeoff in SIP service performance of ≈ 8-ms while ensuring session confidentiality and integrity.
3. It explores the advantages of using multicore and multi-threading options as an alternative to duplicating the entire server hardware.

The remainder of this paper is organized as follows. Section 2 sheds some light on the background of SIP, while Section 3 describes related work. Section 4 defines the proposed model and its implementation, as well as discusses cases related with and without security protocols. Section 5 discusses the performance of SIP with M/M/1 and M/M/c models with and without security primitives. Section 6 describes limitations and challenges, while Section 7 compares our work with others. Finally, Section 8 presents the conclusion and future work.

## 2 Background

The purpose of this study is to introduce readers to the basics of the SIP protocol, M/M/1 and M/M/c queues, and wireless technology.

### 2.1 SIP architecture and its components

**Fig 1:**
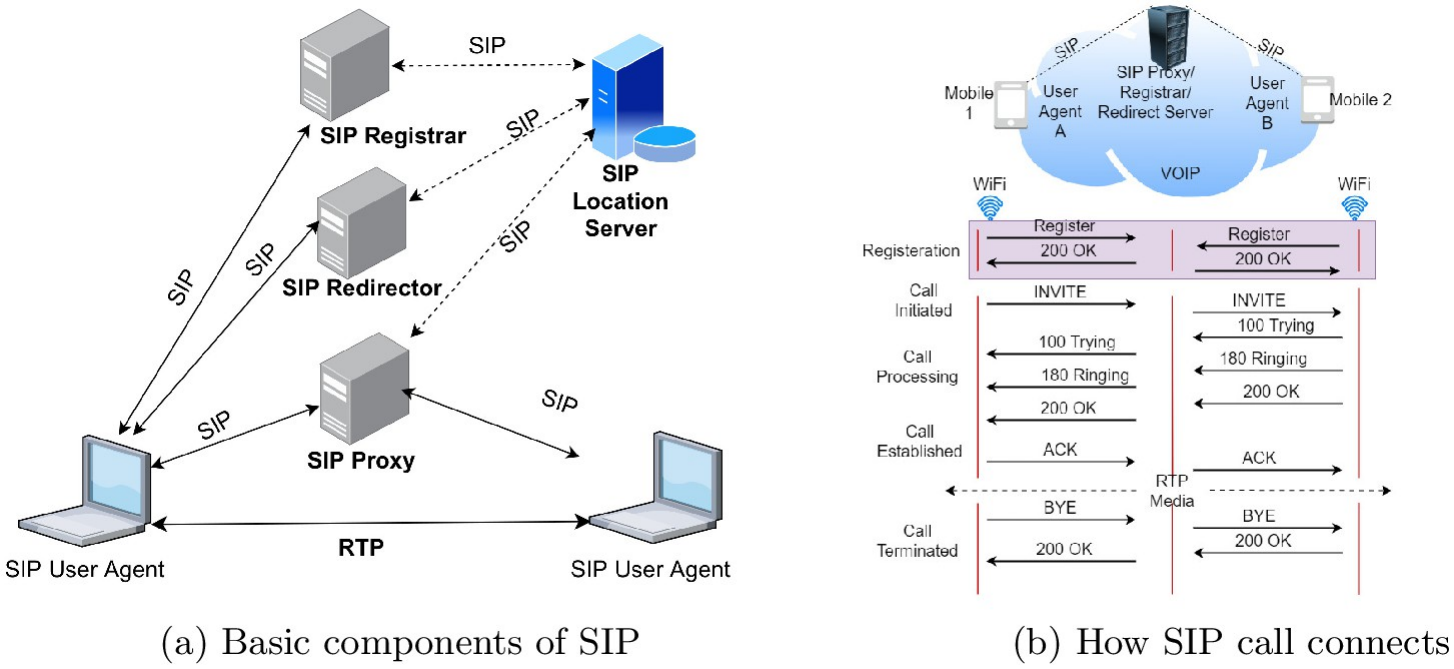
SIP. (a) Basic components of SIP, (b) How SIP call connects.

SIP is a conventional session initiation protocol broadly utilized to control audio and video correspondence meetings over IP. It can be used to create, change, and end unicast (two-party) or multicast (multiparty) meetings comprising one or a few media streams [18, 19]. The essential components of the SIP network are illustrated in Fig 1a, and depicts various types of servers and agents involved in the SIP protocol. The device that generates a request is called the user agent client (UAC), and the device that receives a call is called the user agent server (UAS). SIP servers have predefined syntaxes and semantics for handling requests originating from clients. For example, a proxy server sends a request from a client to another proxy server if the exact address of the recipient is unknown; otherwise, it sends a request to the recipient. The redirect server sends a request to the originator indicating to choose another route to reach the recipient. Users of the SIP network register themselves (locations/IP addresses) with the registrar and periodically refresh it. The location server stores all the locations (SIP/IP addresses) of the SIP users [20]. After switching on the SIP UAC and UAS devices, their IP addresses and capabilities were registered in the SIP registration server (registrar). Since SIP is a message-based signaling protocol, messages are exchanged between UAC and UAS as requests and responses [21, 22]. The key functions and services of SIP include name mapping and redirection, capability negotiation and management, and participant management [23, 24]. The generic SIP REQUEST and RESPONSE messages used for the call/messaging session setup, communication, and teardown are given in Tables 1 and 2, respectively. The remaining control messages can be reviewed from SIP RFCs or online resources such as [25].

**Table 1:** Examples of SIP messages.

Message	Description
“REGISTER”	Registers address given [To] header field.
“INVITE”	Invites a client to participate in a call.
“ACK”	Client has received a response against INVITE.
“BYE”	Ends Meeting
“CANCEL”	Cancels any pending request.
“MESSAGE”	Transports instant messages using SIP.

**Table 2:** Examples of SIP responses.

Code	Code Meanings	Example
1xx	“Informational”	“100” → “Trying”
2xx	“Success”	“200” → “Ok”
3xx	“Redirection”	“305” → “Use Proxy”
4xx	“Client Error”	“400” → “Bad Request”
5xx	“Server Error”	“503” → “Service Unavailable”
6xx	“Global Failure”	“600” → “Busy Everywhere”

When the SIP UAC and UAS devices turn on their IP addresses, information regarding their availability is stored on the SIP registration server. When a UAC hopes to spread out a media meeting with the UAS, it sends a message “INVITE,” requesting the middle server (SIP Proxy) to communicate with UAS. The middle server requests and acquires the IP address of the UAS and other control information from the SIP registration server. It pings UAS using the message “INVITE” and sends “Trying” message to UAC, meaning the proxy is trying to reach UAS. UAC listens with a ringing tone. If UAC’s receives “OK” message, it means UAS is ready to receive data. UAC then sends an “ACK” to UAS. After the connection is successfully established, control is provided to the RTP/SRTP protocol for SIP data communication. Thus, media meetings between the UAC and UAS were established. To end the communication, anybody can send a “BYE” message. The SIP session setup and teardown are illustrated in detail in Fig 1b.

### 2.2 Queuing models

The M/M/1 and M/M/c queuing models have been used in various applications [26–31]. They are used in this study to analyze the behavior of SIP protocols in the presence and absence of application-layer security in WLAN environments. Therefore, this research is limited to M/M/1 and M/M/c queue models for calculating the efficiency of SIP server using its performance metrics, average response time, queue length, and server utilization (probability of server availability), the mathematical and statistical relationships of which are well documented by Inaki et al. [11], Güne [32] and Gosavi [33]. *M/M/1* framework has remarkable appearances (M/M/1), a solitary server (M/M/1) with outstanding help time (M/M/1) and an endless line (certain M/M/1/∞). Eqs 1, 2, and 3 are used to calculate the queue length (*L*_*q*_), average response time (*W*_*q*_), and server utilization (*ρ*) for an M/M/1 queuing model [34].

$$\begin{array}{c} L_{q}=\frac{\rho^{2}}{\left(1-\rho \right)} \end{array}$$ (1)

$$\begin{array}{c} W_{q}=\frac{L_{q}}{\lambda } \end{array}$$ (2)

$$\begin{array}{c} \rho =\frac{\lambda }{\mu } \end{array}$$ (3)

where λ denotes the call arrival rate and *μ* is the service rate. If *c* is the number of servers that handle the arriving SIP calls, then for M/M/c, the server utilization (*ρ*) can be computed using Eq 4. In the present study, c represents the number of threads utilized to handle user calls while the SIP proxy server is running. Each thread is allocated dynamically to a separate processor core. Each thread provided a unique service rate *μ*. The queue length (*L*_*q*_) can be obtained using Eq 5 and the average response time (*W*_*q*_) using Eq 7 [34].

$$\begin{array}{c} \rho =\frac{\lambda }{c\mu } \end{array}$$ (4)

$$\begin{array}{c} L_{q}=[\frac{Po{\left(\frac{\lambda }{\mu }\right)}^{c}\rho }{c!{\left(1-p\right)}^{2}}] \end{array}$$ (5)

*P*_0_ is the probability of zero callers in the communication system and is computed as follows:

$$\begin{array}{c} P_{0}=\frac{1}{\left[\sum_{m=0}^{c-1}\frac{{\left(c\rho \right)}^{m}}{m!}+\frac{{\left(c\rho \right)}^{c}}{c!(1-\rho )}\right]} \end{array}$$ (6)

$$\begin{array}{c} W_{q}=L_{q}/\lambda \end{array}$$ (7)

### 2.3 WiFi selection for local communication: Why?

**Fig 2:**
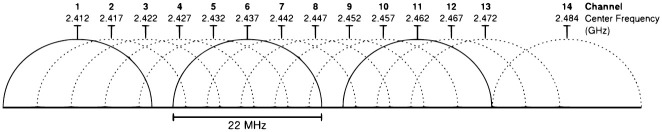
WiFi 2.4 GHz channels.

WiFi is based on the IEEE standard 802.11, which is defined for WLANs. Different version of IEEE 802.11 are IEEE802.11a, IEEE 802.11b, IEEE 802.11 g, IEEE 802.11n, and IEEE 802.11be [35, 36]. In a standard WLAN, a unified switch allows the devices to share WiFi signals operating at 2.4 and 5 GHz frequency bands. IEEE 802.11n is one of the most widely adopted protocols for this purpose. It has a coverage range of 100 m and 14 channels equally spaced within the mentioned bandwidth, as shown in Fig 2. Each channel is 22 MHz and separated by a 3 MHz guard band. This bandwidth was sufficient for audio and video data transmission [37].

The reasons for choosing WiFi include its widespread global adoption across various communication devices, such as mobile phones, smartphones, PDAs, PCs, and laptops. Additionally, WiFi offers high data rates compared to other wireless technologies such as Bluetooth, Zigbee, infrared, and NFC. In addition, it incorporates rate-adaptation and power-management features [38]. The determinants of SIP communication over WiFi include packet loss, jitter, and latency, which are attributed to interference, signal attenuation, and congestion. These issues can compromise call quality and cause delays [39]. WiFi offers the advantages of convenience, mobility, ease of deployment, and continuous technological advancements, making them viable options for addressing the needs of large-scale organizations in the foreseeable future [40, 41].

## 3 Related work

Noori et al. [42] analyzed the efficiency of SIP and H.323 protocols in terms of performance in IEEE 802.11e using the QualNet simulator. They used five metrics: i) total number of bytes used in a session, ii) setup time, iii) bytes sent and received by the RTP, iv) average end-to-end delay, and v) throughput. The authors concluded that the SIP outperformed H.323. However, the study focused solely on performance metrics and did not address security aspects of the protocols. Security is a critical concern for real-world VoIP deployment, and a comparative study should include an analysis of the security features and vulnerabilities of SIP and H.323.

Bahaa et al. [43] worked on VOLTE call failure prediction in SIP-based IMS network using different machine learning algorithms such as KNN, SVM, decision trees, and naive Bayes. The study found that using machine learning, specifically the SVM classifier with wrapper feature selection, achieved a high prediction accuracy (97.5%) for call failures within the IMS network. This method predicts failures and allows preemptive actions. Machine learning also uncovers the causes of call failures, which are difficult to identify through manual tracing. The approach reveals multifactorial causes behind failures in the IMS network, which are vital owing to rising traffic and 5G. Security concerns include data privacy, model vulnerabilities, and adversarial attacks such as model poisoning. Secure deployment and consideration during the development are vital for trust in the network and risk reduction.

Ali et al. [44] demonstrated the effect of block and stream ciphers on SIP, including AES, 3DES, SEED, Camellia, and RC4. Camellia was the most suitable algorithm for low-CPU devices. Similarly, Perigo et al. [45] performed a comprehensive cost-and-benefit analysis of VOIP security performance using VPN, SRTP, TLS, VPN-SRTP, VPN-TLS, SRTP-TLS, and VPN-SRTP-TLS. Their results showed that a VPN with AES and TLS can be implemented without risk compared to other configurations, such as a simple VPN or SRTP. Moreover, SRTP-VPN-TLS is a secure combination; however, troubleshooting is complex.

Febro et al. [46] introduced a protective virtualized network function (VNF) named “microVNF” for safeguarding SIP-based IoT devices. It utilizes edge-switching programmable data planes. MicroVNF defends IoT devices against SIP DDoS attacks before and after infection. Pre-infection thwarts SIP scanning, enumeration, and dictionary attacks. Post-infection obstructs botnet registration to the command-and-control server, preventing command receipts while identifying and countering botnet SIP DDoS attacks. It serves as the first line of defense, blocking malicious packets from reaching the core network and ensuring edge-computing security. Deep packet inspection counteracts dictionary attacks, SIP scanning, and enumeration. However, this study lacks comparative analysis, scalability insights, and assessment against advanced attacks or encrypted traffic. Addressing these gaps and considering real-world deployment will enhance microVNF’s practicality for IoT security.

Montazerolghaem [47] addressed the critical problem of SIP server-resource exhaustion in SIP-based networks arising from distributed routing and network management. Their proposed SDN-based framework aims to optimize SIP server resources in VoIP networks. The framework focused on enhancing throughput and reducing energy consumption. However, the study lacks a thorough security analysis, omitting concerns like vulnerabilities, authentication, data privacy, and protection against typical VoIP cyber threats. The framework failed to ensure secure communication, user authentication, data privacy, and resilience to SIP-based attacks. Adherence to security standards and management interface security was also missing. Enhancing security requires a deeper analysis and implementation of industry best practices.

Mansoor et al. [48] analyzed the performance of SIP protocol in a multimedia system. They used delay, jitter, and packet loss as performance parameters and found that the efficiency of SIP was affected by variations in jitter. However, the research has the following limitations: It does not mirror real SIP networks due to the absence of a gatekeeper. The scope of the project, testing of Open SIP Library apps, and performance analyses were unspecified. Metrics and industry benchmarks other than QoS parameters have been overlooked. The tool choice lacks a thorough explanation. Security analysis is absent, although it is crucial for SIP apps. This study lacks real-world considerations and generalizability, which limits its applicability. A conclusive summary of the areas of improvement is absent.

Diogo et al. [49, 50] proposed to make use of machine- and deep-learning-based approaches for detecting abnormal SIP dialogue sessions. They support their proposal using long short-term memory), recurrent neural networks (RNNs), the Bayesian inference method, and Hidden Markov Model supported by *n*– gram Markov observations. However, the study lacks dataset details affecting result generalizability. The specific vulnerabilities and classifier effectiveness require clarification. A comparative analysis with other methods is missing, which hinders the method assessment. In addition, the absence of false positive/negative rate information limits our understanding of real-world performance. To enhance credibility, additional dataset information, performance comparisons, and robustness analyses are needed.

Fowder conducted a performance analysis of the WebRTC and SIP protocols in [51]. WebRTC provided better-quality video communication than SIP with respect to the signal-to-noise ratio (SNR), whereas SIP outperforms WebRTC in audio transmission in the context of segmental SNR. However, some limitations exist. This study lacks detailed information on the dataset size and diversity, potentially affecting the generalizability of the results. In addition, the SIP vs. WebRTC comparison focuses on specific applications, neglecting varied network conditions and codec options. The evaluation covered only the VP8 and VP9 codes for WebRTC, limiting the representation. A comprehensive assessment of metrics such as latency and scalability is missing. Furthermore, the effect of real-time speech translation on the communication quality remains unexplored.

Mahamood et al. [52] presented an improved authenticated key agreement protocol for SIP proposed by Dongqing et al. [53]. However, the protocol is vulnerable to insider attacks, denial-of-service, and secret leakage. Moreover, it relies on strict time synchronization, which is impractical in real-world settings. Despite these claims, this study lacks thorough evaluation and comparison. To ensure effectiveness and security, wider validation under diverse scenarios is required.

Waleed et al. proposed a DDoS detection scheme based on RNN for SIP-based networks with satisfactory results [54]. Typical SIP authentication is weak due to reliance on basic methods like digest access authentication, lack of multi-factor authentication, and vulnerability to attacks like brute force and spoofing [55, 56]. These shortcomings blur the lines between attackers and legitimate users, increasing DDoS threats. Therefore, conventional SIP authentication struggles to counter malicious actions. However, the study lacks details on the dataset size and diversity, impacting the generalizability of the results. Moreover, the resilience of the model to adversarial attacks and dynamic networks is unexplored. Missing is a comparison with DDoS methods and the evaluation of false positives/negatives, hindering comprehensive assessment. Strengthening credibility and utility requires larger and varied datasets and robustness testing.

Zhou and Chen [57] assessed existing SIP ECC-based authentication schemes and discovered vulnerabilities in Arshad et al.’s [58] and Lu et al.’s [59] schemes. Zhou and Chen proposed an efficient anonymous ECC-based solution that can withstand known attacks. However, comparisons with other schemes and their resilience to adversarial attacks are lacking. Further validation across different scenarios is required to ensure confidence in their effectiveness. Ashraf et al. [60] and Ashraf et al. [61] studied the multimedia SIP performance in IP-based MCCS systems. Load-impacted registration affects the overall performance. Faycal et al. [62] explored SIP in an IMS with an MPLS, demonstrating efficient handling of sessions. NGN supports numerous subscribers while maintaining quality; however, security concerns in this context have not been thoroughly addressed. Noor et al. [63] suggested SIP protocol for IEEE 802.11 networks. However, security implications, crucial for successful communication, were overlooked.

Qiu et al. [64] enhanced the SIP security solution based on the two-factor authentication method proposed by Kumari et al. [65], which are prone to guessing attacks. Furthermore, design issues in the key agreement phase render it less viable. The forward secrecy method proposed in [64] rectified the design issues in [65]. Azeddine et al. [66] analyzed SIP and H.323 performance using MIPv6. SIP surpassed H.323 in terms of call setup efficiency, jitter, end-to-end delay, and packet loss. However, the mobility challenges in MIPv6 were not deeply addressed, and security analysis, which is crucial for sensitive VOIP, was lacking. This gap makes the system vulnerable to attacks and threats.

Goshi [67] conducted a performance analysis of the SIP protocol in IEEE 802.11a and IEEE 802.11b environments. The parameters used to study the performance of SIP included throughput, packet drop rate, and end-to-end delay. However, considering IoT growth, SIP-enabled mobiles could be compromised. While comparing the SIP performance in WLANs using 802.11a and 802.11b, this study shows limitations As it focuses on two radio link standards and excludes others. Metrics such as jitter, congestion, and scalability were overlooked. Security and vulnerabilities in SIP-WLAN deployment have not been addressed and are essential for real-world use.

Sureshkumar et al. [68] employed virtualized network function (VNF) in an edge switch to boost SIP performance. The performance analysis used the M/M/c and M/M/1 queuing models, revealing useful insights. However, this approach may not fully capture the real-world SIP traffic dynamics. Moreover, VNF scalability and real network implementation challenges remain unexplored.

SSL/TLS is a secure protocol that safeguards web traffic through encryption and hashing. Its evolution has addressed vulnerabilities by utilizing handshakes and digital certificates for secure connections. However, SSL is unsafe because of vulnerabilities such as raccoon attacks. Moreover, TLS 1.3 is recommended over insecure versions such as pre-TLS 1.2 [69]. In SIP, SSL/TLS enhances security through encryption and authentication, ensuring private data exchange between clients and servers. HTTPS is enabled by SSL/TLS for websites. By adding S/MIME and IPSec to SIP, security is bolstered against threats, such as eavesdropping and unauthorized access. However, the limitations include SSL/TLS stripping and man-in-the-middle attacks. HTTP digest in SIP provides entity authentication but lacks mutual authentication, data integrity, and confidentiality. Moreover, weak passwords expose offline dictionary threats. The TLS’s public key infrastructure degrades SIP performance, and SRP-TLS is vulnerable to offline dictionary attacks. S-SIP offers an alternative to TLS. S/MIME encrypts SIP packets but adds processing overhead. IPSecs at the OS level is often unsupported by SIP clients, complicating the infrastructure [7, 70].

### 3.1 Problem definition

From the literature review, it was observed that SIP, a widely used VOIP signaling protocol, loses its security when deployed without HTTPS (TLS/SSL) over the Internet. IPSec and S/MIME have their limitations (overhead and infrastructure) that do not permit their use because they may deteriorate the performance of SIP-based communication. In WLAN scenarios, these protocols are unnecessary. Because HTTPS (SSL/TLS) is meant for Internet-based secure communication, ensuring the end-to-end security of the SIP protocol in a WLAN scenario becomes the responsibility of application developers. Very little work has been done on WLAN-based SIP implementations that establish secure audio, video, and text communication using WLAN connectivity and analyze it under different queuing systems. While SIP performance has been examined in various scenarios, to the best of our knowledge, its performance on multi-threaded SIP servers in WLAN setups with different queuing models has not been investigated, especially with and without customized end-to-end security measures. This perspective of SIP communication is studied in this research and may completely replace old PBX/PABX or fixed VOIP phone sets.

## 4 Proposed framework

Our dependence on wired technology for communication has been ingrained well, but its practicality diminishes over time owing to inherent limitations and unreliability. Hence, wired LANs are widely being replaced with WLANs due to higher repair times. We adopt a more advanced wireless SIP framework instead of the old PBX/PABX as a viable solution for messaging, meetings, and calls because it enables highly secure seamless voice and video data v, offering a more reliable and efficient alternative. The present study focuses on using SIP based on various boundaries to assess the improved outcomes in WLAN setups.

**Fig 3:**
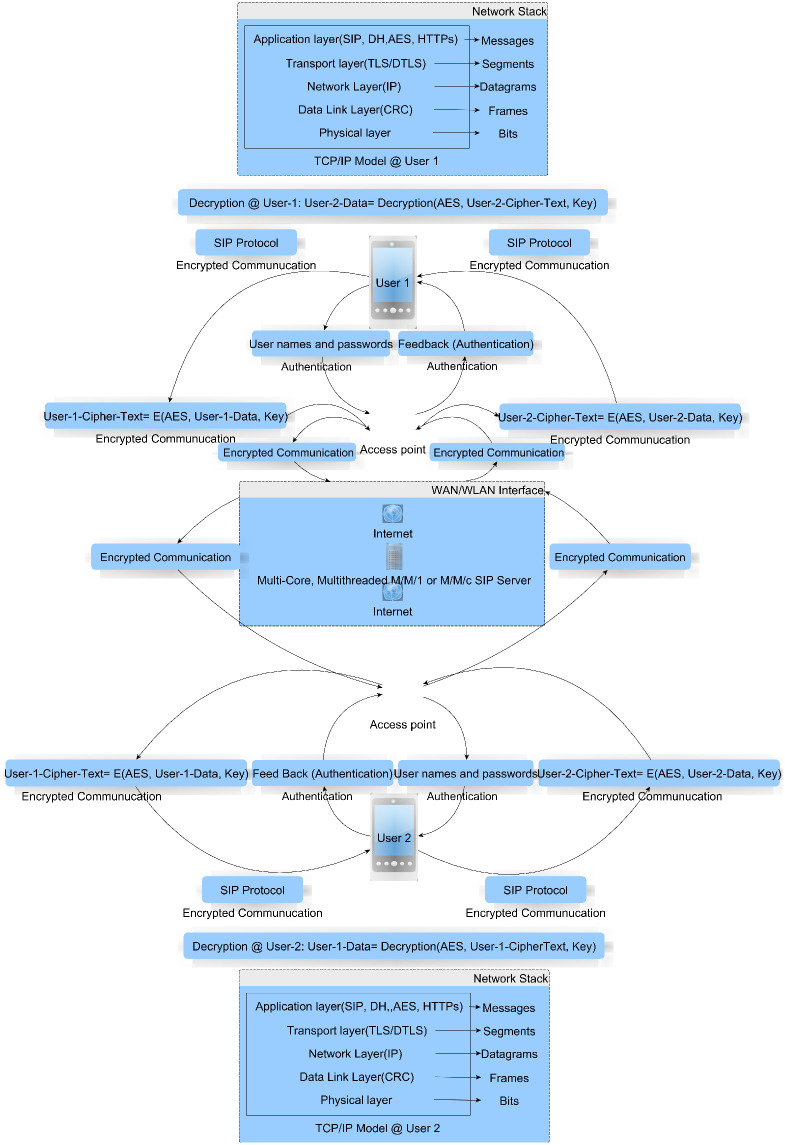
Proposed model.

WiFi 802.11 is the WLAN network standard to link two mobile devices for secure communication using WPA, WPA2, and WPA3. WEP is obsolete and no longer used. When SIP communication is established over the internet, HTTPS ensures security; otherwise, no security is provided. In the present scenario, we developed local-area communication using SIP instead of PBX/PABX. Therefore, no security provisions are available at the application layer. For data protection at the application layer, we ensure symmetric cryptography-based security at the SIP application layer in a WLAN environment to ensure confidentiality, integrity, and authentication [71]. The effectiveness of the proposed framework is assessed by measuring the parameters, such as average response time of a call in a queue, queue length, and server utilization in terms of occupancy, while eliminating complete reliance on an internet connection as compared to applications such as Skype, Yahoo Messenger, WhatsApp, and Vchat. The proposed framework is illustrated in Fig 3.

We implement the SIP architecture to encrypt the messages between two devices: UAC and UAS. UAC is an endpoint that may begin, alter, and terminate sessions, such as encrypted instant chats or voice conversations. In the transport layer, TLS is the security measure used by SIP to avoid transport-layer attacks. Using credentials, user-1 (UAC) authenticates itself, and the plain message is encrypted using a shared secret into ciphertext before transmission, which is then passed through a proxy server to user-2 (UAS). This is similar to how the router receives a request from the user agent and transmits it to another user.

### 4.1 Key exchange

**Fig 4:**
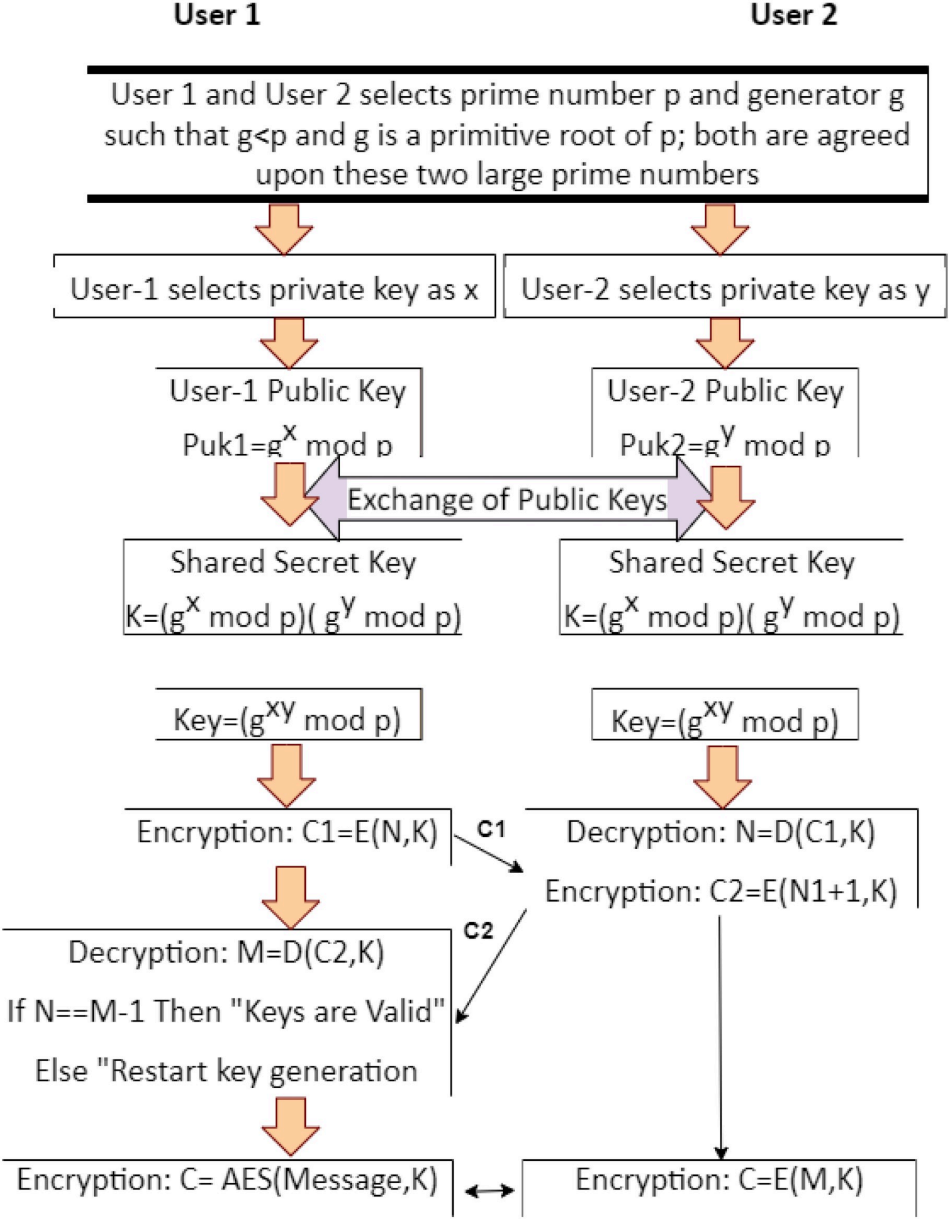
Secret key acquisition process.

We did not write any protocols for key exchange; rather, we modified the readily available secure Diffie-Hellman (DH) key exchange for exchanging security keys [72]. In the proposed key acquisition process called modified DH, two or more users generate their private and public keys; public keys of the users are shared first, and a secret key is generated using these shared public keys [73]. This approach enhances security and is commonly used in cryptographic protocols to establish secure communication channels between parties because it is fully compatible with symmetric and asymmetric key cryptography [74]. In Fig 4, user-1 and user-2 generate their private and public keys. Both users exchange public keys using a modified DH protocol, after which each user calculates an identical secret key at their respective end. This secret key is then used for the encryption and decryption of textual and multimedia streams. In this study, although numerous low-cost encryption methods, such as LED, PRESENT, and SIMON, are available [75], we chose the globally standardized and widely supported AES encryption algorithm [76]. PRESENT and LED support smaller key sizes than AES, whereas SIMON offers a key size equivalent to AES. AES is widely optimized for hardware, and SIMON performs better than AES in software implementation. SIMON supports larger block sizes for data encryption than AES and can be used for resource-constrained IoT devices [77]. However, it is less tested and supported in industry and may not receive the same level of support and integration compared to AES. In contrast, AES is a well-established, thoroughly tested, and widely adopted encryption standard in many industries, and numerous libraries and hardware implementations support it [78]. Therefore, we use AES rather than LED, PRESENT, or SIMON.

#### 4.1.1 Pairwise key exchange

As shown in Fig 3, user-1 and user-2 choose a prime number *p* and a generator *g* such that *p* > *g*, and *g* is a primitive root of *p*. Subsequently, they select private key values *x* and *y*. User-1 sends its public key, *g*^*x*^ *mod* *p*, to user-2, and user-2 sends its public key, *g*^*y*^ *mod* *p*, to user-1. Both parties calculate their complete secret keys using the formula *key* = *g*^*xy*^ *mod* *p*. At this point, the generated keys are symmetric and can be used for secure communication.

#### 4.1.2 Group key exchange

For group communication, we assume there is a third user, user-3. If user-3 is also involved in the key exchange process, it sends its public key *g*^*z*^ *mod* *p*, provided that it selects *z* as its private key. It also receives *g*^*x*^ *mod* *p* and *g*^*y*^ *mod* *p* from user-1 and user-2. Thus, all three users compute *g*^*xyz*^ *mod* *p* as their group keys.

Present security solutions are computationally secure, but with sufficient resources, they can be breached. There are information-theoretic cryptography solutions that are resilient against the large computing power of an adversary [79], such as quantum key distribution and secret key generation using information-theoretic perspectives of wireless physical or MAC layer characteristics for use in the future [80].

User-2 decrypts the message sent by user-1 using a shared secret key; the encrypted reply is sent to user-1 through the proxy server, which decrypts with the same shared secret key. SIP resides between the mobile device and proxy server and provides secure communication through an IEEE 802.11 access point. The Internet interface is optional for a local WLAN communication setup but is required for global WAN communication. The authentication mechanism is based on usernames/passwords and is similar to the classical schemes proposed in [81, 82].

### 4.2 Setup

1. *SIP Proxy Server:* Open SIP Express Router (OpenSER), written in C Programming language, was selected for testing. A standard process-based concurrency control was implemented in this proxy with shared memory for sharing the state of concurrent calls. It has been proven quite efficient when multicore processors handle multi-threading. Each thread is run in a single microprocessor core, and further thread scheduling improves the efficient use of the processor. OpenSER has good documentation and industry support across the globe.
2. *User Database:* MYSQL is one the most reliable and light DBMS used for medium size database systems. Because it is a free open-source database, it is used for creating and maintaining user databases. Users are authenticated using user names and passwords; enhanced authentication can also be seen from [64].
3. *Call Generator:* SIPp call generator is used, but in this study, we developed a call generator on Android mobile; 2 to 20 clients were asked to make calls simultaneously. Thus, simultaneous calls were generated at the rate of 2–20 calls/min.
4. *Operating System:* Microsoft Windows 10 for SIP Proxy Server and Android OS for a client is selected in this study. We used Android Pie 9.0 on mobiles and Microsoft Windows 10 on Dell PC. Android Pie 9.0 is an open-source working framework having a graphical UI (GUI) for ease of use.
5. *Hardware*: 11th Generation core i5 Dell laptop with 2.40 GHz processor and 8 GB RAM is used. The system has four cores. Thread scheduling is performed such that a single thread handles no more than three calls each of one minute. Thus, the hardware with 8 cores is fine for testing. However, no more than 6–7 threads are created.
6. *DH Key Exchange:* Users generate their private and public keys; public keys are exchanged through the DH key exchange protocol over a common channel, which is also accessible to the attacker. However, the attacker gains no benefit even if it captures the public key. Each user computes shared secret using a modified version of the DH protocol; then, the shared key is used to encrypt text, audio, and video streams using AES. Symmetric key encryption is faster than asymmetric, making AES preferable over RSA [83].

### 4.3 Implementation

This section elaborates on the proposed security framework and observes its behavior with and without end-to-end encryption in the M/M/1 and M/M/c queue systems. The call arrival and service rates are random and can be considered a Poisson process. This result implies that the number of incoming calls at any given interval follows a Poisson distribution, which is a discrete probability distribution. The distribution is used to find the chance of an event occurring a certain number of times within a given period and has a single parameter λ, indicating the mean number of events (the call arrival rate in this study). Eq 8 provides the probability of *x* calls in a unit period [84].

$$\begin{array}{c} \begin{array}{cc} P(x) & =\frac{e^{-(\frac{call}{time}*time\;interval)}{\left(\frac{call}{time}*time\;interval\right)}^{x}}{x!}\\ & =\frac{e^{-\lambda }\lambda^{x}}{x!} \end{array} \end{array}$$ (8)

In the above equation, *e* is the Eulier number, whose value is 2.71828; it is an important constant used in many applications for determining the growth or decay factor over time. We used the M/M/1 and M/M/c queuing models such that the total number of calls in the session could be processed through a queue. For this, the calls originate following a random process such as Poisson, which has already been considered. During this session, key metrics, such as the average number of calls, average response time, and server utilization, were computed to determine a superior queuing model for different SIP scenarios based on their performance. Symmetric key cryptography is the primary security mechanism in our framework. It assures privacy and authentication at the application layer and is the most preferable framework for securing audio/video calls and messaging.

The simple call connection procedure is shown in Fig 1b, and the complete proposed SIP is given in Algorithm 1. Two implementation scenarios are considered: Studying SIP using queuing models (i) without and (ii) with encryption protocols. The algorithm establishes secure SIP connections using AES encryption and Diffie-Hellman secret keys. This supports multi-threading to handle multiple SIP connections. It also introduced two queuing models, M/M/1 and M/M/c, to simulate server processing. If the number of calls exceeded a certain threshold, M/M/c was used; otherwise, M/M/1 was used. This algorithm ensures secure data transmission and connection teardown.

**Algorithm 1** Secure SIP Connection Establishment and Data Transmission with AES Encryption (Multithreading)

1: **Initialization**: Choose a large prime *p* and a primitive root *g* for the Diffie-Hellman key exchange.

2: **Global Variables**: connections ← []

3: server_capacity ← *c*    ▹ Number of servers in the MMC queue

4: **User A**: secret_key_A ← GenerateRandomNumber()

5: **User B**: secret_key_B ← GenerateRandomNumber()

6: **User A**: public_key_A ← *g*^secret_key_A^ mod *p*

7: **User B**: public_key_B ← *g*^secret_key_B^ mod *p*

8: **Connection Establishment**:

9: **procedure** SIP_Connect()

10:  **User A** → **User B**: [public_key_A, AES_encrypted(SIP_Request)]

11:  **User B** → **User A**: [public_key_B, AES_encrypted(SIP_Response)]

12: **Shared Secret Key Generation**:

13: **User A**: shared_secret_A ← CalculateSharedSecret(*g*, *p*, public_key_B, secret_key_A)

14: **User B**: shared_secret_B ← CalculateSharedSecret(*g*, *p*, public_key_A, secret_key_B)

15: **Data Transmission**:

16: **procedure** SIP_Data_Transfer()

17:  **User A** → **User B**: AES_encrypted(SIP_Data, shared_secret_A)

18:  **User B** → **User A**: AES_encrypted(SIP_Data, shared_secret_B)

19: **Connection Teardown**:

20: **procedure** SIP_Disconnect()

21:  **User A** → **User B**: AES_encrypted(Teardown_Request, shared_secret_A)

22:  **User B** → **User A**: AES_encrypted(Teardown_Response, shared_secret_B)

23: **M/M/1 Queuing Model**:

24: **procedure** M/M/1_Queue()

25:  **User A** → **Server**: SIP_Request

26:  **Server**: Process SIP request

27:  **Server** → **User A**: SIP_Response

28: **M/M/c Queuing Model**:

29: **procedure** M/M/c_Queue()

30:  **User A** → **Server**: SIP_Request

31:  **Server**: Assign server for processing SIP request

32:  **Server** → **User A**: SIP_Response

33: **Main Function**:

34: **procedure** Main()

35:  connections ← [SIP_Connect, SIP_Connect, …, SIP_Connect]    ▹ Add as many connections as needed

36:  calls_threshold ← *T*   ▹ Threshold value for switching to M/M/c

37:  total_calls ← length of connections

38:  **if** total_calls > calls_threshold **then**

39:   connections ← [M/M/c_Queue, M/M/c_Queue, …, M/M/c_Queue]    ▹ Use M/M/c if calls exceed threshold

40:  **else**

41:   connections ← [M/M/1_Queue, M/M/1_Queue, …, M/M/1_Queue]    ▹ Use M/M/1 if calls are below threshold

42:  Start all threads in connections concurrently

43:  Wait for all threads to complete

### 4.4 Scenario-1: Study of SIP by using queuing models without encryption protocols

This scenario observes how SIP behave when their calls are stored and processed using M/M/1 and M/M/c queues in the absence of end-end security primitives.

#### M/M/1

**Fig 5:**
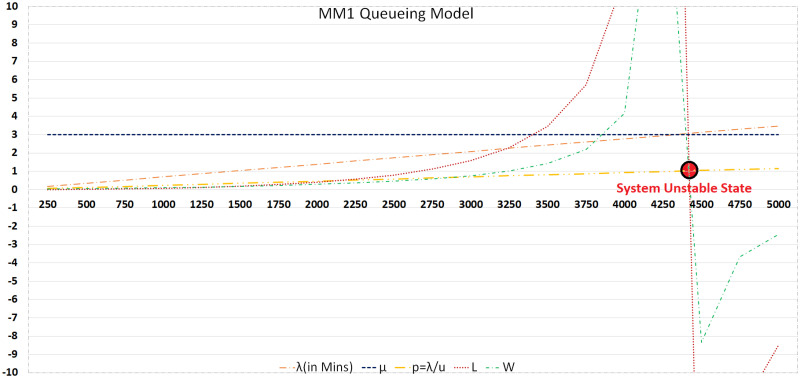
M/M/1 queuing model.

When the number of calls (λ) rises to 4500 calls/day (3.125 calls/min), the communication system becomes unstable at a service rate (*μ*) being 3, as shown in Fig 5. The probability of the server being free (1>*ρ*>0) detects this unstable state. When *ρ* > 1 (1.041666), the system has reached an unstable state. This means that at this call and service rate, a single server queuing model can only handle 4250 calls/day, which is unsuitable for most large business organizations.

#### M/M/c

**Fig 6:**
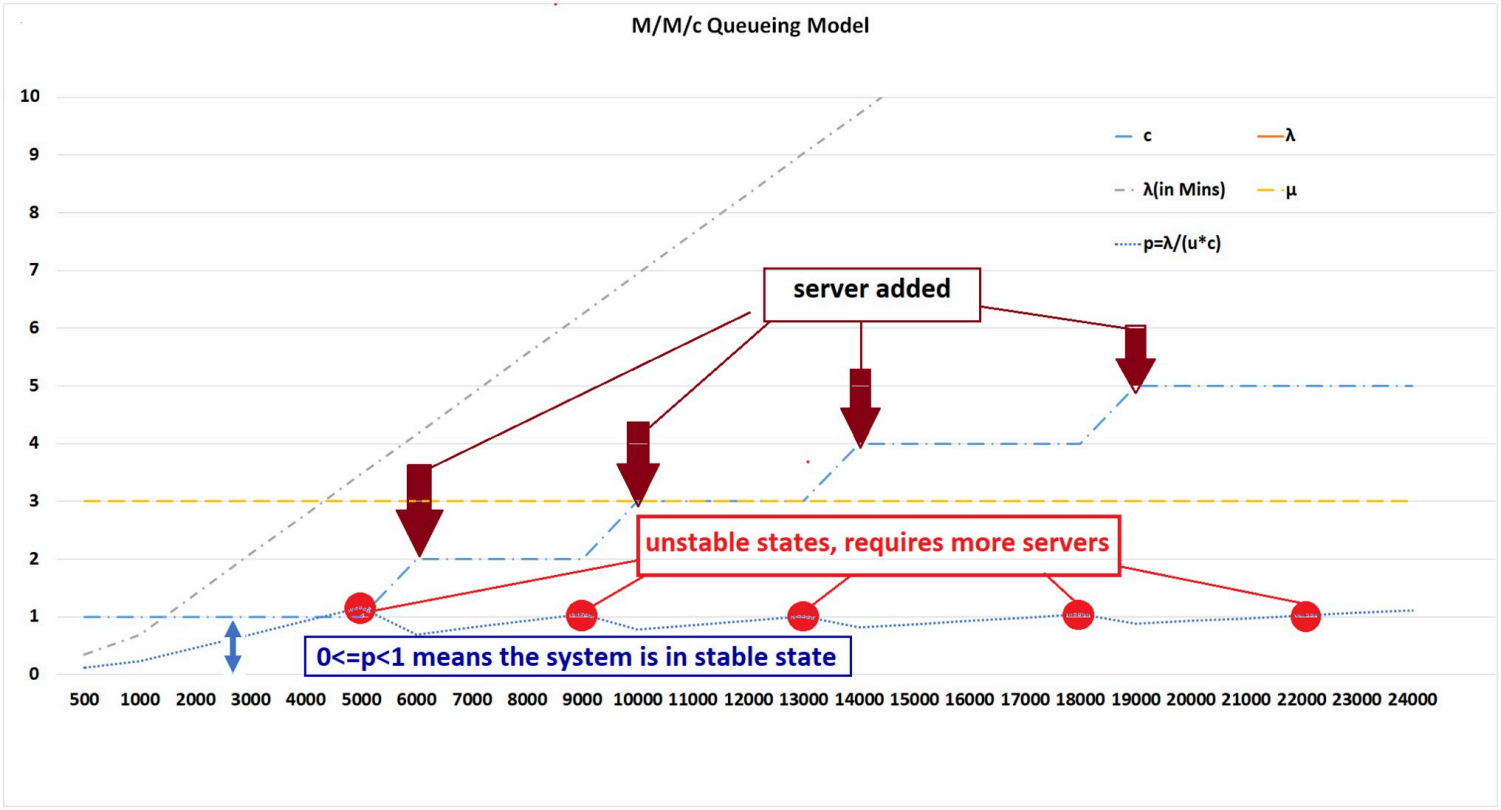
M/M/c queuing model.

From the M/M/1 discussion, a more fair queuing model is M/M/c should be considered for a larger number of calls. Instability indicates that more servers are required, which can be analyzed using M/M/c. Fig 6 provides a detailed analysis of M/M/c-based queue systems and demonstrates how systems become unstable and can be restored to normal operation by adding more servers. The system becomes unstable when the call rate goes beyond 2.951 calls/min (4250 calls/day) in the case of a single server. In the case of two servers, the system exhibited stability when the call rate was 5.902. Similarly, the system is stable with 3, 4, 5, and 6 servers at a call rate less than or equal to 6.07, 8.85, 11.97, 14.93, and 17.88, respectively.

## 5 M/M/c: SIP performance with and without security primitives

**Fig 7:**
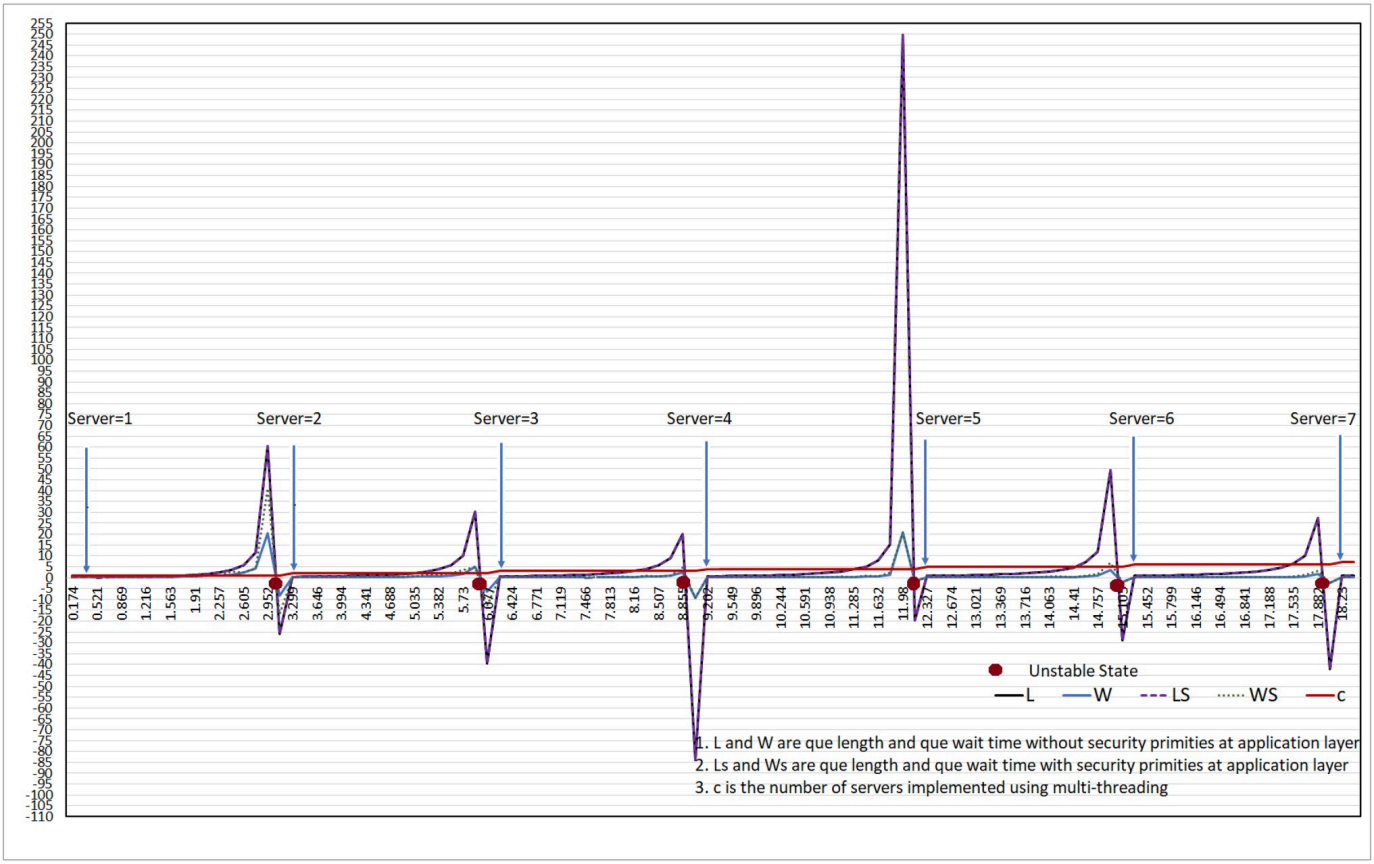
W, L of M/M/c without end-end security.

This section discusses the performance of the M/M/c-based SIP protocol with and without security primitives. We consider three parameters: the number of calls/min (call arrival rate: λ), call wait time (*W*_*q*_) in the queue, and the number of calls in the queue (*L*_*q*_). The results of the M/M/c queue length and wait time with and without application-layer security primitives are shown in Fig 7 with the number of servers varying from 1–7.

### 5.1 Call wait-time in queue

The wait time of incoming SIP calls in the queue is expected to increase when using security primitives at the application layer. The average increase in wait time (in min) in the queue is 0.658357, 0.513328, 0.494961, 0.46810, 0.167469, 0.217606, and 0.246230 when the number of servers is 1, 2, 3, 4, 5, 6, and 7, respectively. However, the benefits of adding security outweigh the increase in the wait time.

### 5.2 Queue length: Number of calls in queue

The length of the queue increases as the number of calls/day increases. However, adding more servers to the system helps balance the effect of the increased number of calls in the queue. Despite servers equipped with security measures handling slightly fewer calls than those without security measures, the difference is not significant when considering the benefits of maintaining security. On average, when using application-level security in the SIP system, the number of calls served was slightly lower for each server. For example, a system with 1, 2, and 3 servers serves approximately 2.05, 2.08, and 2.28 fewer calls compared to a corresponding system without security measures. However, the tradeoff of implementing added security is considered worthwhile.

### 5.3 Server availability

**Fig 8:**
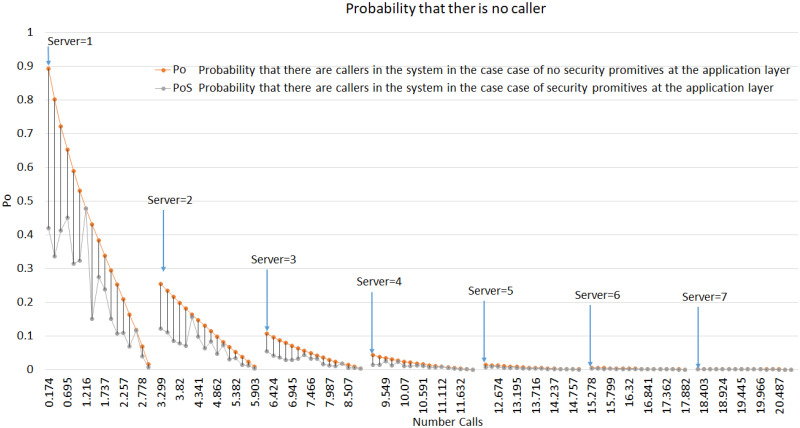
Probability that there is no callers.

**Fig 9:**
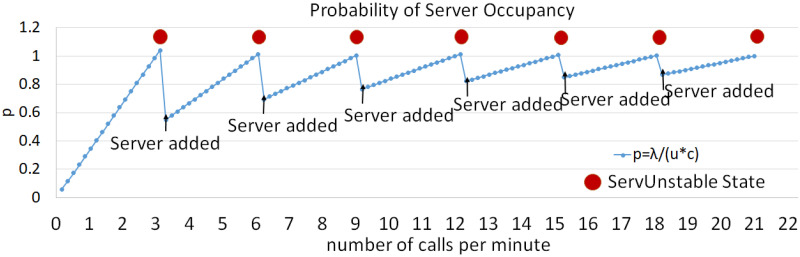
Server occupancy.

When the server is burdened with additional security overhead, its capacity to handle more calls is compromised, increasing the number of callers waiting in the queue. Analyzing server availability helps determine the number of calls required to add a server when security protocols are involved. Although the effect is expected to be negligible, a more robust analysis of the SIP behavior is possible. Fig 8 shows the probability of no caller in the queue with and without security primitives. This shows that the probability of no callers decreases with increased number of servers. A decline in the mentioned probability observed with server/s 1, 2, 3, 4, 5, 6, and 7 is 16.92%, 0.79%, 0.49%, 0.28%, 0.058%, 0.003%, 0.0053%, respectively. The values decrease because the number of servers and the call rate increases. The values may vary in different tests owing to the random behavior of the communication system; however, the ability to serve more calls is increased by adding new servers to the system, as shown in Fig 9. Fig 9 shows that the server occupancy varies according to the call rate and server numbers. Moreover, the likelihood of a server being unavailable increases as the number of calls increases. However, when the system enters an unstable state, it starts dropping calls. By introducing an additional server, the system resumes its stable state, and the probability of server occupancy decreases.

### 5.4 *M*/*M*/1 vs *M*/*M*/*c*

In the absence of end-to-end security primitives at the application layer, at a service rate of 3 calls, the response time of *M*/*M*/1 is 0.00355 min at 250 calls/day and 20.23809 min at a maximum load of 4250 calls/day (2.95 calls/min). The system becomes unstable after this call arrival rate. The results are the same for M/M/c for the same number of servers but vary with increasing number of servers. However, if the number of servers increases from one to two, the worst-case probability of a server not being available at the maximum arrival rate (i.e., 4250 calls/day or 2.95 calls/min) is 0.492, the number of calls in the queue would is 0.23825, and the response time achieved is 0.08070 min, which is acceptable. However, this acceptance is also determined by an organization’s acceptance threshold policy. By introducing an additional server, the number of calls entertained is seven times greater than the number of calls served with one server at a response time of 0.02126 min. In addition, by increasing the number of servers from one to two at the maximum call arrival rate (i.e., 4250 calls/day or 2.95 calls/min), the queue length decreased from 60 to 0.23825, and wait time from 20 to 0.08070 min. Similar gains in server availability, response time, and queue length can be obtained by adding 2, 3, 4, 5, or 6 servers. The studies recommend that at a service rate of 3 calls/min, it is better to adopt the M/M/c model if the call arrival rate is more than 2500 calls/day, as the response time is approximately 0.45 min and the number of calls in the queue is 0.75. However, the response time was no longer acceptable after 2500 calls/day at the mentioned service rate.

### 5.5 Effect of AES and security achievements

**Fig 10:**
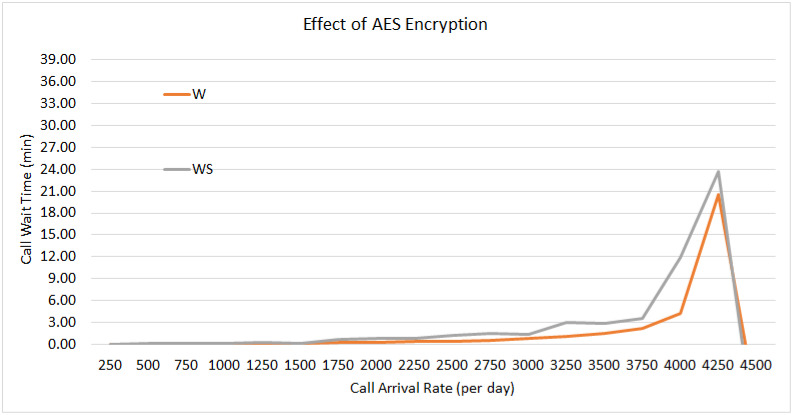
Effect of AES.

In the absence of encryption, the call experiences a delay of approximately 32 ms, which increases to approximately 40 ms with encryption. SIP, due to its inherent nature, lacks security. The implementation of secure media streaming and dependable signaling to preserve the confidentiality and integrity of the exchanged session setup data incurs a modest tradeoff of 8 ms in SIP service performance. Fig 10 represents a zoomed-in segment of Fig 7, highlighting a slight gap between “W” (waiting time without encryption) and “WS” (waiting time with encryption).

## 6 Limitations and challenges

Presently, not all mobile and smartphones support advanced versions of IEEE 802.11, 802.11ac, 802.11ax, and 802.11be, having Gbps speeds. Furthermore, a small number of employees do not have smartphones, making it impossible to install SIP applications on them. Therefore, it may be difficult to deploy the solution at a large scale and harness the true capacity of IEEE 802.11. Similarly, despite being better than AES, we could not propose the SIMON block cipher because it is new and industry support is currently minimal. Some employees do not have network extensions in their offices or have to go to another office to make or receive calls. In real-world scenarios, when all employees are provisioned to make or receive calls using their smartphone or mobile devices, the results may vary slightly from the simulation results. The real capacity of WiFi IEEE 802.11 can only be obtained if employees without network extensions are removed and all smartphones are equipped with the latest WiFi version.

## 7 Comparison with related studies

The proposed work was compared with related studies that presented different perspectives on SIP. The results are summarized in Table 3. Most literature has focused on the evaluation and performance of SIP. Some studies have focused on the local (WLAN)-based implementation of the SIP protocol. Although calling systems such as VOIP/SIP are queuing-based, most researchers have ignored SIP performance assessments using a queuing system. Similarly, most SIP assessments relied on traditional security primitives. However, our work used SIP in a local environment independent of the Internet connection and WiFi connectivity to establish communication and reduce the cost of Internet usage. Moreover, this study evaluated SIP in M/M/1 and M/M/c queue models, proposed a customized security-based DH public key exchange, and acquired secret keys for AES encryption.

**Table 3:** Comparison of related studies on SIP.

Study	Based [WAN/WLAN]	Security Primitives	Encryption	Multi-core/Multi-threading feature	Limitation	Queuing Model
Noori et al. [42]	WLAN	No	NO	NA	does not address security	No
Ali et al. [44]	WAN	TLS, encryption algorithm user dependent	AES, 3DES, SEED, Camellia, and RC4	NA	AES consumes 1.9% of CPU usage RC4 has low throughput and runs with insufficient throughput due to stream cipher behavior	No
Febro et al. [46]	Edge Computing/IoT	Virtualized network function (application-layer firewall and intrusion-detection) called “microVNF” to protect SIP-based IoT devices using programmable data plane feature on the edge switch against SIP DDoS		NA	lacks comparison with other security solutions; evaluation of microVNF against sophisticated attacks or encrypted traffic is not addressed	No
Mahamood et al. [52]	WLAN, WAN	improvement of Dongqing et al. [53] and proposed authenticated key agreement		NA	i) prone to privileged insider attacks, DOS, and ephemeral secret leakage attack, ii) strict assumption of time-synchronized system does not advocate environments where time synchronization cannot be guaranteed	No
Perigo et al. [45]	WAN	AES, TLS, VPN,SRTP	AES	NA	performance analysis of SIP with different configurations	No
Waleed et al. [54]	Independent	DDoS detection	Authentication protocol (Digest access authentication)	NA	conventional authentication methods like digest access authentication are weak as they lack multi-factor authentication (MFA)	No
Zhou and Chen [57]	WLAN, WAN	Proposed ECC based authentication protocol		NA	i) Encrypted messages are bigger in size; ii) chances of implementation errors due to the complexity of ECC algorithms; iii) ECC is less secure in quantum computers as they require fewer qubits to decode [85]	No
Ashraf et al. [60], Ashraf et al. [61], Faycal et al. [62]	WAN/GSM	NA	NA	NA	focused only on the performance of IMS systems and does not discuss security aspects except negligible discussion by Ashraf et al. [61]	No
Sulafa et al. [86]	WAN	IPSec, TLS, S/MIME, DTLS, and HTTP digest authentication	AES	NA	performance analysis without considering queuing models or capabilities of multicore, multi-threaded processors	No
Qiu et al. [64]	IoTs, WSNs, Ad Hoc Networks, etc.	two-factor authentication, private key cryptography		NA	i) design issues in key agreement phase makes [64] less viable; ii) [65] is prone to guessing attack; iii) no performance analysis conducted	No
Noor et al. [63]	WLAN	No	NO	NA	i) polling-based medium access increases overhead as the size polls/coordinators increase; ii) this study does not address security aspects	No
Satanu Goshi [67]	WLAN	NA		NA	i) Performance analysis only	No
Sureshkumar et al. [68] (VNF)	WLAN	Virtualized Network Function		NA	scalability and practical implementation of VNF in real networks may require further investigation and consideration	M/M/1, M/M/c
Proposed	WLAN	Customized, Simple	AES	Multi-core, Multi-threaded	every employee need not have a smartphone and latest WiFi version	M/M/1, M/M/c

## 8 Conclusion and future work

Studies have suggested the diminishing practicality of wired communication technology owing to its limitations and unreliability. Therefore, an advanced wireless SIP-based framework and a suitable queuing system are needed for more efficient and reliable messaging, meetings, and calls. Software-based solutions, such as multithreading instead of duplicating hardware-based servers, can handle multiple call requests simultaneously. Moreover, instead of duplicating the servers, the multicore technology of modern computers can be used. This involves running multiple threads across cores of the same computer/server to manage concurrent call requests, significantly reducing hardware costs. Our study reveals that the M/M/1 queuing model works well when the calls are less than 3200/day as the wait time is 1 min; however, the waiting time increased to 20 min at 4250 calls/day, after which the system halted. Therefore, the SIP should transition to M/M/c if the call rate surpasses a threshold. Our model could manage ≈ 8250, 12750, 17250, 21250, and 25750 calls/day using 2, 3, 4, 5, and 6 threads, respectively, each operating on separate computer system cores. These results were presented without thread scheduling. The call service rate can be increased using scheduled multi-threading. The customized end-to-end security provision causes negligible loss in SIP performance. Moreover, implementing secure media streaming and reliable signaling for session confidentiality and integrity with AES incurs a minor tradeoff of ≈ 8-millisecond in SIP service performance.

In the future, we will extensively investigate of the SIP protocol considering various input parameters. These parameters include the number of individuals and calling devices available within an organization. Notably, we observed scenarios where the number of calling devices was insufficient compared to the number of individuals, leading to device sharing for making calls. We believe that exploring such a communication system model will yield valuable insights.

## Supporting information

S1 File (XLSX)

S1 Data (CSV)

S2 Data (CSV)
